# How much does vaccination reduce the rate of HBV infection in Iranian population? a Bayesian adjustment analysis 

**Published:** 2019

**Authors:** Sajad Shojaee, Farid Zayeri, Maryam Nasserinejad, Ali Ghasemzadeh, Saeedeh sadat Beheshti Shirazi, Mahsa Khodadoostan

**Affiliations:** 1 *Gastroenterology and Liver Diseases Research Center, Research Institute for Gastroenterology and Liver Diseases, Shahid Beheshti University of Medical Sciences, Tehran, Iran.*; 2 *Department of Biostatistics, Faculty of Paramedical Sciences, Shahid Beheshti University of Medical Science, Tehran, Iran.*; 3 *Basic and Molecular Epidemiology of Gastrointestinal Disorders Research Center, Research Institute for Gastroenterology and Liver Diseases, Shahid Beheshti University of Medical Sciences, Tehran, Iran*; 4 *Department of Gastroenterology and Hepatology, Faculty of Medicine, Isfahan University of Medical Sciences, Isfahan, Iran*

**Keywords:** Vaccination, Hepatitis B virus, Misclassification, Underreporting, Bayesian adjustment

## Abstract

**Aim:**

The aim of this research was to estimate the changing rate of odds ratio (OR) by varying degrees of hepatitis B virus (HBV) underreporting.

**Background:**

Data registering is usually associated with extensive errors such as misclassification, under-reporting, missing data due to lack of co-operation, error prone factors, and in medical studies, inadequate diagnosis of physicians or low accuracy of laboratory tests. In the present study, which discuss the actual impact of vaccination on HBV prevention, exposure and response were prone to various errors. Furthermore, some people in the community are possibly infected to the virus while were not reported in the count of patients with HBV infection.

**Methods:**

This was a case control study. Cases included patients with HBV referring to the gastroenterology and liver disease research center. The control group included patients without HBV who underwent a fatty liver test at Taleghani hospital laboratory. Bayesian approach and Gibbs sampling algorithm were used to estimate OR.

**Results:**

According to results, misclassification rate was mild in raw data, but with an increase in degree of underreporting for 50 and 500 of unreported cases, OR increased by about half and more than double, respectively, while sensitivity diminished strikingly.

**Conclusion:**

Our analysis asserted that knowing the degree of underreporting is essential to accurately calculate OR and sensitivity. In addition, despite varying OR in different samples, overall the results were similar according to the pattern of exposure and response association.

## Introduction

 Hepatitis B is a viral infection and a common cause of liver disease and cancer (1,2). Hepatitis B vaccine has become available since 1982. Nevertheless, chronic HBV infection has remained one of the major global problems for public health and a main cause of deaths from cirrhosis and liver malignancy in the world (3–5). Although the spread of the virus infection in developed countries is relatively low, it is still prevalent in eastern and Southeast Asia (6–8).

The hepatitis B immune coverage increased from the beginning of 2000 with the support of the global union for vaccination and immunization (9). The national vaccination program for hepatitis B virus for all newborns and high-risk groups began in 1993 in Iran. Since then, all newborn babies have been covered by the vaccination program and receive the vaccine in three stages at regular intervals: at birth, 1.5 months after birth, and 9 months after birth. Thus, the epidemiological pattern of the prevalence of hepatitis B virus has changed over time in Iran, and a reduction has been reported in both the acute and chronic infection of the virus (10).

Iran is a medium-risk country with a prevalence of 2-7% and nearly 2 million adults with chronic infection. In another meta-analysis study (11), the prevalence of hepatitis B virus infection in the Iranian population was 2.2%. The highest prevalence of the virus was reported in Golestan province with 8.9% while the lowest in Kermanshah province with 0.7%. The prevalence of the virus was 2.9% before 2010, while after 2010 the prevalence dropped to 1.3%.

In clinical and epidemiological studies, to investigate the association between a binary exposure and response, the nature of these types of studies can often lead to misclassification due to the low accuracy of diagnostic tests. Because of the close association between the accuracy of the test and its sensitivity as well as specificity (12), misclassification is defined as a function of sensitivity and specificity for exposure and response.

One of the most important reasons for misclassification is the lack of a gold standard. Thus, by changing the definitions of classification, misclassification occurs. When clinical and laboratory data are available, this issue can be mitigated. Thus, clinical researchers can reduce the classification error according to the data. On the other hand, when data is not available, statistical techniques can be used. One of these techniques is the Bayesian method (13).

In a study that is prone to misclassification for both exposure and response; non-differential response correction can be matched by three models using the hierarchical Bayesian approach. Since the present study is a public vaccination and has been proceeded for a few decades, vaccination information may have a recall bias, and HBsAg marker can be associated with a slight error. Consequently, misclassification and unreported cases affect HBV sensitivity and Odds Ratio. 

We applied Bayesian adjustment to obtain the real effect of the vaccination on hepatitis B virus and to estimate the validity as well as accuracy of the intensity of effect despite misclassification error and underreporting cases. 

## Methods

**Source and study population**

This was a case control study in which both cases and controls were prone to misclassifications. Cases include those suspect to have hepatitis B virus referring to the gastroenterology and liver disease research center at Shahid Beheshti University of Medical Sciences by its vice chancellor in health affair. The control group included those who underwent a fatty liver test at Taleghani hospital laboratory and they were not suspect to carry hepatitis B virus.

**Modeling**

Exposure variable of the present study was being up to date in 3 times of communal vaccination and response variable was hepatitis B virus infection. Correction of exposure and response values was performed based on the assumption of exposure and response misclassification in accordance with the exposure misclassification approaches of Gustafson (14) and Luta et al. (15) via three models. These three models include exposure model (equation 1), measurement model (equation 2), and response model (equation 3), adjusted for appropriate covariates (in related studies (16–19), risk factors associated with HBV were selected) as follows:

$\mathrm{logit}\left\{P\left(\mathrm{UTD}=1\right)\right\}=\alpha_{0}+\alpha_{1}\mathrm{Age}+\alpha_{2}\mathrm{Ethnicity}+\alpha_{3}\mathrm{Surgury}+\alpha_{4}\mathrm{Cupping}$

(1)

$p\left({\mathrm{case}}^{*}\right)=r\left(\mathrm{SN}\right)+\left(1-r\right)\left(1-\mathrm{SP}\right)$

(2)

$\mathrm{logit}\left\{r=P\left(\mathrm{Case}=1\right)\right\}=\beta_{0}+\beta_{1}\mathrm{UTD}+\beta_{2}\mathrm{Age}+\beta_{3}\mathrm{Ethnicity}+\beta_{4}\mathrm{Surgury}+\beta_{5}\mathrm{Cupping}$

(3)

**Priors/Validation data**

In order to implement the analysis in the Bayesian framework, it is necessary to specify the distribution of the parameters and the prior probabilities. In the exposure and response models, we used independent informative normal priors for the intercepts, covariates, as well as the up-to-dateness in the hepatitis B vaccine. Also, for the sensitivity (SN) and specificity (SP) in the measurement model, as prior distributions, it is required to calculate the number of reported cases and non-cases. We let A represent a true positive, B a false positive, C a true negative, and D a false negative. Also, E and F represent the number of unreported cases and controls in the remaining population, respectively. The values of the sensitivity priors changed subsequently with varying several degrees of underreporting, though the specificity remained almost perfect. Thus, for sensitivity and specificity, we used beta distribution with appropriate parameters as follows: for sensitivity, beta (A, C + E); and for specificity, beta (D + F, B). Precision estimates are provided as 95% confidence intervals and 95% credible limit ratios to facilitate comparisons.

**Bayesian framework**

In order to correct the values of exposure and response, we first consider correction of exposure values by the exposure model. The imperfect response values are also corrected using the beta distribution for sensitivity and specificity in the measurement model. Since the Bayesian inference of the posterior equation in logistic models (equations 1 and 3) will be in a complex form and create multi-dimensional density functions (20), the inferences were performed via Morkov Chain Monte Carlo simulation and Gibbs sampling algorithm.

**Resampling**

The present study population reflects the situation of Iran on a small scale (0.01% of the total Iranian population). Indeed, Iran is in the middle risk zone with a prevalence of about 2%. The prevalence of HBV infection was estimated to be 2.14 and 2.7 in the review studies by Alavian et al. (21) and Porolajal et al. (22), respectively. Therefore, assuming a study population of 8000, a sample of 300 was taken from the 3000 available data. Then, in order to determine the impact of unreported cases on sensitivity and subsequently the odds ratio, we changed the unreported cases to 0, 5, 50 and 500. For more accurate investigation of the pattern of changes in OR and misclassification rate and also to avoid random occurrences, the results were interpreted based on three-time resampling. Thus, at each step of the simulation, using 1000 Gibbs iterations and excluding 50% of the initial observations for burn-in, the correct state of vaccination and imperfect measure of HBV were imputed from the exposure and measurement model and regressed in the outcome model to form a posterior distribution of the estimates of the corrected odds ratios. Empirically, only simulations that improve the likelihood were retained to estimate the posterior distribution. The analysis was performed using R, version 3.5.1. 

## Results

**Demographic changes of available data in the present study**

The frequency distribution of the 2000 reported cases and 1000 reported controls is reported in Table 1. Overall, 79% of the population were not up-to-date on their vaccination (n=2364), of whom 72% were cases (n=1694). Also, about half of the population was female (50%), mostly over 30 years (79%) and were married (86%). Compared to the control, most cases were from other ethnicities (65% vs. 50%), non-cupping (82% vs. 64%), and non-surgical (52% vs. 42%). Nevertheless, there was no significant difference for the incidence of smoking (18% vs. 13%) and alcohol use (5% vs. 4%).

**Table 1:** Demographic changes of characteristics of the study and comparison of reported potential cases to controls

Characteristic	Total No.	Total %	Case No.	Case %	Control No.	Control %
Sex	3000		2000		1000	
Man	1515	50.5	1056	52.8	459	45.9
Woman	1485	49.5	944	47.2	541	54.1
Age	3000		2000		1000	
<=30	642	21.4	588	29.4	54	5.4
>30	2358	78.6	1412	70.6	946	94.6
Marriage	3000		2000		1000	
Not Married	416	13.9	299	15.0	117	11.7
Married	2584	86.1	1701	85.1	883	88.3
Education	3000		2000		1000	
Illiterate	404	13.5	374	18.7	30	3.0
Not Illiterate	2596	86.5	1626	81.3	970	97.0
Ethnicity	3000		2000		1000	
Fars	1187	39.6	692	34.6	495	49.5
Others	1813	60.4	1308	65.4	505	50.5
Cupping	3000		2000		1000	
Yes	710	23.7	351	17.6	359	35.9
No	2290	76.3	1649	82.5	641	64.1
Tattooing	3000		2000		1000	
Yes	341	11.4	167	8.4	174	17.4
No	2659	88.6	1833	91.7	826	82.6
Periodontal	3000		2000		1000	
Yes	1774	59.1	1534	76.7	240	24.0
No	1226	40.9	466	23.3	760	76.0
Sexuality	3000		2000		1000	
Yes	23	0.8	17	0.9	6	0.6
No	2977	99.2	1983	99.2	994	99.4
Shaving	3000		2000		1000	
Yes	386	12.9	330	16.5	56	5.6
No	2614	87.1	1670	83.5	944	94.4
Bloodslashing	3000		2000		1000	
Yes	114	3.8	56	2.8	58	5.8
No	2886	96.2	1944	97.2	942	94.2
HBsAg	3000		2000		1000	
Positive	2000	66.7	2000	100.0	0	0.0
Negative	1000	33.3	0	0.0	1000	100.0
HBsAb	3000		2000		1000	
Positive	366	12.2	5	0.3	361	36.1
Negative	2634	87.8	1995	99.8	639	63.9
HBcAb	3000		2000		1000	
Positive	2094	69.8	2000	100.0	94	9.4
Negative	906	30.2	0	0.0	906	90.6
HcvAb	3000		2000		1000	
Positive	2957	98.6	1961	98.1	996	99.6
Negative	43	1.4	39	2.0	4	0.4
Vaccination	3000		2000		1000	
Yes	636	21.2	306	15.3	330	33.0
No	2364	78.8	1694	84.7	670	67.0
Surgery	3000		2000		1000	
Yes	1541	51.4	966	48.3	575	57.5
No	1459	48.6	1034	51.7	425	42.5
Smoking	3000		2000		1000	
Yes	490	16.3	363	18.2	127	12.7
No	2510	83.7	1637	81.9	873	87.3
Alcohol	3000		2000		1000	
Yes	157	5.2	94	4.7	63	6.3
No	2843	94.8	1906	95.3	937	93.7
Drug abuse	3000		2000		1000	
Yes	198	6.6	179	9.0	19	1.9
No	2802	93.4	1821	91.1	981	98.1

**Prior specification of HBV sensitivity and specificity: Sample One**

Of the 8,000-person population, 300 were reported cases and controls, while the remaining 7700 were non-reported in the community. Due to the lack of confirmed laboratory and clinical data for correcting classification of cases and controls, based on the simulation framework and knowledge to a low error rate of laboratory tests, 197 were correctly identified as true case patients with 5 false positives while 96 were correctly identified as true non-case patients with 2 false negatives. Assuming a complete case report (E = 0, no underreporting), this corresponds to 99% sensitivity and near perfect specificity. Afterward, by altering the degree of underreporting (E = 5, 50, 500), sensitivity diminished to 97%, 79%, and 29%, respectively (Table 2). In addition, the existence of valid data and unnecessary need for expert opinion creates the following set of priors: 

${\mathrm{SN}}_{E=0}\mathrm{\sim beta}(197,2)$, ${\mathrm{SP}}_{E=0}\mathrm{\sim beta}(7796,5)$, ${\mathrm{SN}}_{E=5}\mathrm{\sim beta}(197,7)$, ${\mathrm{SP}}_{E=5}\mathrm{\sim beta}(7791,5)$, ${\mathrm{SN}}_{E=50}\mathrm{\sim beta}(197,52)$, ${\mathrm{SP}}_{E=50}\mathrm{\sim beta}(7746,5)$, ${\mathrm{SN}}_{E=500}\mathrm{\sim beta}(197,502)$, ${\mathrm{SP}}_{E=500}\mathrm{\sim beta}(7296,5)$

**Prior specification of HBV sensitivity and specificity: Sample Two**

As in the first example, Of the 8,000-person population, including 300 reported cases and controls, 181 were correctly identified as true case patients with 7 false positives while 106 were correctly identified as true non-case patients with 6 false negatives. Assuming a complete case report (E = 0, no underreporting), this corresponds to 97% sensitivity and near perfect specificity. Afterward, by varying the degree of underreporting (E = 5, 50, 500), sensitivity declined to 94%, 76%, and 26%, respectively (Table 3). In addition, we have the following set of priors: 〖SN〗_(E=0)~beta(181,6), [P〗_(E=0)~beta(7806,7), 〖SN〗_(E=5)~beta(181,11), 〖SP〗_(E=5)~beta(7801,7), 〖SN〗_(E=50)~beta(181,56), 〖SP〗_(E=50)~beta(7756,7),〖SN〗_(E=500)~beta(181,506), 〖SP〗_(E=500)~beta(7306,7)

**Prior specification of HBV sensitivity and specificity: Sample Three**

As in previous examples, Of the 8000-person population, including 300 reported cases and controls, 197 were correctly identified as true case patients with 4 false positives while 95 were correctly identified as true non-case patients with 4 false negatives. Assuming a complete case report (E = 0, no underreporting), this corresponds to 98% sensitivity and near perfect specificity. Afterward, by varying the degree of underreporting (E = 5, 50, 500), sensitivity dropped to 96%, 78%, and 28%, respectively (Table 4). In addition, we have the following set of priors: 

${\mathrm{SN}}_{E=0}\mathrm{\sim beta}(197,4)$,                    ${\mathrm{SP}}_{E=0}\mathrm{\sim beta}(7795,4)$,                 ${\mathrm{SN}}_{E=5}\mathrm{\sim beta}(197,9)$, ${\mathrm{SP}}_{E=5}\mathrm{\sim beta}(7790,4)$,                 ${\mathrm{SN}}_{E=50}\mathrm{\sim beta}(197,54)$,                 ${\mathrm{SP}}_{E=50}\mathrm{\sim beta}(7745,4)$, ${\mathrm{SN}}_{E=500}\mathrm{\sim beta}(197,504)$, ${\mathrm{SP}}_{E=500}\mathrm{\sim beta}(7295,4)$

**Bayesian Adjustment for correcting bias**

**Table 2:** Specification of prior distribution of HBV sensitivity and specificity as beta distribution by assuming varying degrees of underreporting in the first sample

Calculation	Specification	Scenario 1: E=0			Scenario 2: E=5			Scenario 3: E=50			Scenario 4: E=500		
		No.	a	b	No.	a	b	No.	a	b	No.	a	b
Total population	A+B+C+D+E+F	8000			8000			8000			8000		
Reported population	A+B+C+D	300			300			300			300		
Cases	A+B	202			202			202			202		
non-cases	C+D	98			98			98			98		
Disease Classification													
True positives	A	197			197			197			197		
False positives	B	5			5			5			5		
False negatives	C	2			2			2			2		
True negatives	D	96			96			96			96		
Non-reported	E+F	7700			7700			7700			7700		
non-cases	F	7700			7695			7650			7200		
Underreported cases	E	0			5			50			500		
Sensitivity	A/(A+C+E)	0.99			0.97			0.79			0.29		
Beta distribution	(A,C+E)		197	2		197	7		197	52		197	502
Specificity	(D+F)/(B+D+F)	1.00			1.00			1.00			1.00		
Beta distribution	(D+F,B)		7796	5		7791	5		7746	5		7296	5

**Table 3:** Specification of prior distribution of HBV sensitivity and specificity as beta distribution by assuming varying degrees of underreporting in the second sample

Calculation	Specification	Scenario 1: E=0			Scenario 2: E=5			Scenario 3: E=50			Scenario 4: E=500		
		No.	a	b	No.	a	b	No.	a	b	No.	a	b
Total population	A+B+C+D+E+F	8000			8000			8000			8000		
Reported population	A+B+C+D	300			300			300			300		
Cases	A+B	188			188			188			188		
non-cases	C+D	112			112			112			112		
Disease Classification													
True positives	A	181			181			181			182		
False positives	B	7			7			7			6		
False negatives	C	6			6			6			5		
True negatives	D	106			106			106			107		
Non-reported	E+F	7700			7700			7700			7700		
non-cases	F	7700			7695			7650			7200		
Underreported cases	E	0			5			50			500		
Sensitivity	A/(A+C+E)	0.97			0.94			0.76			0.26		
Beta distribution	(A,C+E)		181	6		181	11		181	56		181	506
Specificity	(D+F)/(B+D+F)	1.00			1.00			1.00			1.00		
Beta distribution	(D+F,B)		7806	7		7801	7		7756	7		7306	7

**Table 4:** Specification of prior distribution of HBV sensitivity and specificity as beta distribution by assuming varying degrees of underreporting in the third sample

Calculation	Specification	Scenario 1: E=0			Scenario 2: E=5			Scenario 3: E=50			Scenario 4: E=500		
		No.	a	b	No.	a	b	No.	a	b	No.	a	b
Total population	A+B+C+D+E+F	8000			8000			8000			8000		
Reported population	A+B+C+D	300			300			300			300		
Cases	A+B	201			201			202			202		
non-cases	C+D	99			99			98			98		
Disease Classification													
True positives	A	197			197			197			197		
False positives	B	4			4			4			4		
False negatives	C	4			4			4			4		
True negatives	D	95			95			95			95		
Non-reported	E+F	7700			7700			7700			7700		
non-cases	F	7700			7695			7650			7200		
Underreported cases	E	0			5			50			500		
Sensitivity	A/(A+C+E)	0.98			0.96			0.78			0.28		
Beta distribution	(A,C+E)		197	4		197	9		197	54		197	504
Specificity	(D+F)/(B+D+F)	1.00			1.00			1.00			1.00		
Beta distribution	(D+F,B)		7795	4		7790	4		7745	4		7295	4

**Table 5:** Estimated magnitude of naive and Bayesian-corrected OR by assuming varying degree of underreporting in the first to third samples

Sample 1	Analysis and Outcome Measure	OR	95% CrI	95% CrI Ratio
	Naïve			
	Basis measurement	3.14	1.65 - 6.05	3.66
	Bayesian correction			
	No underreporting	3.22	1.96 - 5.37	2.74
	5 True cases not reported	3.42	2.08 - 5.7	2.74
	50 True cases not reported	4.67	2.8 - 7.92	2.82
	500 True cases not reported	6.8	3.96 - 12.03	3.04
Sample 2	Naïve			
	Basis measurement	3.51	1.88 - 6.71	3.58
	Bayesian correction			
	No underreporting	3.75	2.28 - 6.25	2.74
	5 True cases not reported	3.94	2.38 - 6.62	2.78
	50 True cases not reported	5.15	3.06 - 8.83	2.88
	500 True cases not reported	7.24	4.14 - 13.07	3.16
Sample 3	Naïve			
	Basis measurement	3.37	1.76 - 6.56	3.73
	Bayesian correction			
	No underreporting	3.54	2.17 - 5.85	2.7
	5 True cases not reported	3.74	2.27 - 6.24	2.75
	50 True cases not reported	5.1	3.05 - 8.69	2.85
	500 True cases not reported	7.32	4.25 - 12.95	3.04

In the first to third samples, the odds ratios were estimated with raw data. These estimates serve as a basis for comparison, with values of 3.14, 3.51, and 3.37, respectively. After correcting misclassification using the Bayesian approach and the Gibbs sampling algorithm, ideally (i.e. E=0), OR increased to 3.22, 3.75, and 3.54, respectively. As can be seen in Table 5, as the degree of underreporting increased, the adjusted OR increased dramatically. In addition, the credible intervals ratios in the corrected state were smaller than in the misclassification state. The autocorrelation functions and time series graphs of β_1_ estimates in the first to third samples for various degrees of underreporting were obtained using 1000 Gibbs iterations and 50% burn-in. In the following, the graphs related to the first sample are presented.

**Figure 1:**
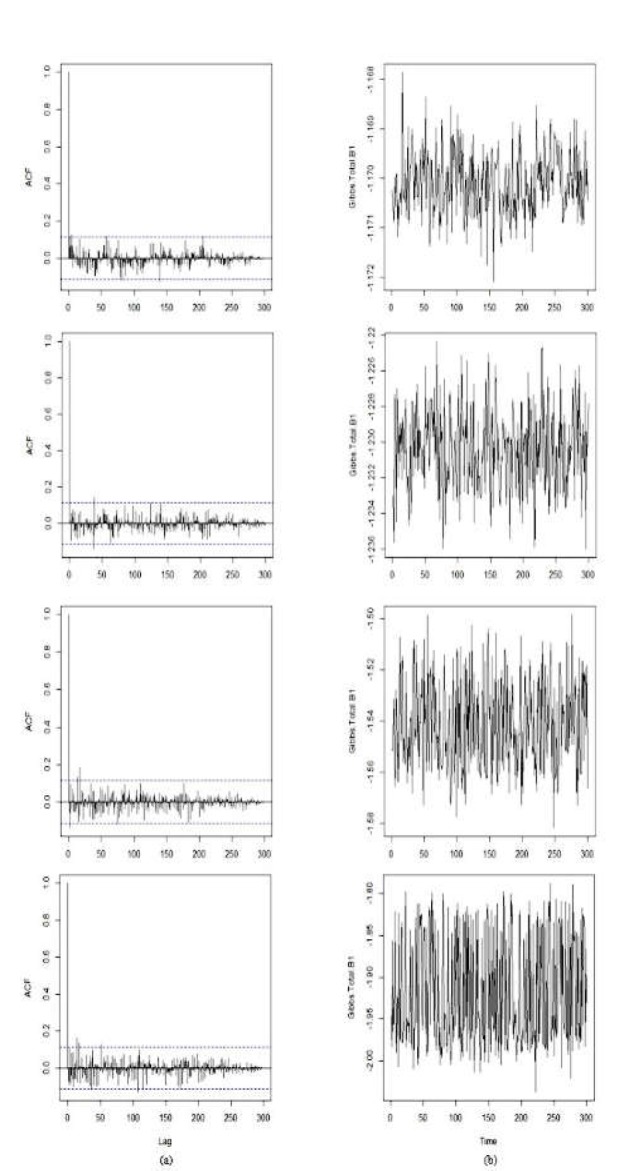
*Autocorrelation Functions (a) and Time Series(b) Graphs of* *β*_1_ *Estimates in the First Sample*

## Discussion

The results of the current research indicated that the misclassification rate was mild in the raw data. The odds ratio increased by only a few percent when only 5 persons of rest of the population in the study population were infected with the virus. On the other hand, for 50 and 500 persons of rest of the population, OR estimates increased by about half and more than double, respectively. Another result of our analysis was that despite the varying OR in the samples, the overall results (misclassification rate in raw data, magnitude of increased effect size in scenarios, etc.) were identical according to the pattern of association (association of exposure and response).

In the present study, there are several reasons for the misclassification of vaccination exposure. Indeed, a group of population may be hesitant to receive the vaccine and over time they experience a recall bias. Another group may not be up to date on the vaccination and has received the vaccine less than three times. Another group may have received the vaccine after being infected with hepatitis B virus, in which case the injected vaccine is dysfunctional on the prevention of the virus. Also, HBV infection is diagnosed with serologic markers including HBsAg, which may be associated with a slight error due to inadequate accuracy of laboratory test which leads to the misclassification of patients with Hepatitis B virus and as a result biased the odds ratio in the present research.

Underreporting of HBV and HCV has long been recognized as a challenge. In the study by Robert et al. (23), the accuracy of state surveillance case registries for recording clinically-confirmed cases of HBV and HCV infections was investigated in few reports. The study indicated that chronic hepatitis B and C went unreported in Pennsylvania Department of Health (PDoH), as compared to patients in the Geisinger Health System of Pennsylvania. In addition, 28% underreporting of HBV and HCV co-infections in the study may lead to biased results about estimation of liver cancer risk (24–26). Another study tested the completeness of report of clinically-confirmed cases of chronic HBV and HCV infections in Michigan, which was unreported with 18% for HBV and 35% for HCV. The difference of unreported cases with demographic characteristics in HBV infection was significant only in the year of initial diagnosis (27).

In a similar study by Goldstein et al. (13) on the impact of vaccination on pertussis disease that was prone to both exposure and response error, the odds ratio and subsequent vaccine efficacy (VE) were corrected; compared to the misclassification state, such as our analysis, no significant difference was observed. Also, varying the degree of underreporting resulted in a change in sensitivity from 90% to 20% and VE estimate from 57% to 82%, while in our study, OR estimates was greater with varying the degree of underreporting. Perhaps one reason is because of the inherent association between vaccination and hepatitis B virus. In another study, Luta et al. (15) in the state of misclassification and missing data biases, with a Bayesian method and similar to our study method, compared four models: A model composed of misclassification and missing data biases at the same time, two models each with one of these biases, and a one raw model. They found that despite the different odds ratios across the four models, overall results were similar with respect to the pattern of associations.

To the best of our knowledge, there was no study examining misclassification of HBV infection rate despite public vaccination. Also, most of the studies investigated the effect of misclassification on either exposure or outcome; however, our study simultaneously evaluated the effects of misclassification on both exposure and outcome through the model and compared the resulting effects.

One of the limitations of the current research was lack of data on proper injection of HBV dose for different ages as well as adherence to the cold chain (28). Secondly, for convenience, we assumed non-differential misclassification. Possibly, by varying the degree of underreporting, cases might have been grouped at an older age causing differential misclassification. Finally, due to the lack of a similar study accurately analyzing our method and subject matter, we were forced to use priors of similar studies. Nevertheless, prior competencies were confirmed during the implementation of the program.

Our study indicated that in retrospective studies of the vaccine and a specific disease/infection associated misclassification error, the actual magnitude of the association has become illusory and the results are underestimated with increasing unreported cases. One solution to this problem is the Bayesian method. In addition, although data from this study were obtained from Taleghani Hospital in Tehran, patients had participated in this study from different cities of Iran. Thus, we can think about generalizing the results to the Iranian society. Finally, the method used in this research can be applied to any arbitrary exposure and response that is prone to misclassification error.

## Conflict of interests

The authors declare that they have no conflict of interest.
